# Integrity and Quantity Evaluation of Plasma Cell-Free DNA in Triple Negative Breast Cancer

**Published:** 2019

**Authors:** Mahdieh Salimi, Somayeh Sedaghati Burkhani

**Affiliations:** Department of Medical Genetics, Institute of Medical Biotechnology, National Institute of Genetic Engineering and Biotechnology (NIGEB), Tehran, Iran

**Keywords:** Cell-free DNA, Real-time PCR, Triple negative breast neoplasms

## Abstract

**Background:**

Triple-Negative Breast Cancer (TNBC) is a subtype of breast cancer that lacks expression of the estrogen and progesterone receptor and does not overex-press human epidermal growth factor 2 receptor protein. TNBC is associated with special characteristics, including aggressiveness, poor prognosis, and treatment response. Non-invasive blood-based molecular markers such as cell-free DNA (cfDNA) variables have been shown to be putative markers in breast cancer prognosis.

**Methods:**

The cfDNA quantity and integrity were assessed in a case-control study of 96 breast cancer patients including 46 triple negative and 50 non-triple negative compared with 50 unaffected controls. A quantitative real-time PCR approach based on the quantification of two amplicons of the *β-actin* gene with different lengths (99 and 394 *bp*) was used to evaluate the integrity index 394/99.

**Results:**

Both cfDNA integrity index and quality were significantly elevated in breast cancer patients but integrity index can be considered as the more reliable diagnostic marker. The statistically significant increase of cfDNA quantity and integrity was observed in TNBC patients, somehow associated with nodal metastasis (p<0.001).

**Conclusion:**

Elevated cfDNA concentration and integrity index in breast cancer patients compared with normal control and significant difference observed between TNBC and non-TNBC may be considered as a possible effective non-invasive diagnostic and prognostic molecular marker in breast cancer.

## Introduction

The Progesterone Receptor (PR), Estrogen Receptor (ER), and Human Epidermal growth factor Receptor 2 (HER2) are well known predictive markers in breast cancer. The Triple-Negative Breast Cancer (TNBC) is classified as a group with no expression of ER, PR, and HER2, accounting for 15–26% of all breast cancer patients. TN breast cancer cases usually show poor prognosis and treatment response 1.

The terms “triple negative” and “basal-like” are not completely synonymous 2. The word triple negative refers to the immunohistochemical classification, whereas the basal-like subtype is defined *via* gene expression microarray analysis.

Circulating cell-free DNA (cfDNA) as tumor-derived fragmented extracellular DNA provides a non-invasive, personalized genomic snapshot of a patients' tumor and has huge potential in prenatal diagnosis 3, disease surveillance, and tumor diagnosis 4. Quantification and assessment of cfDNA have emerged to be a possible tool for early diagnosis of cancers, which has been confirmed in the variety of cancers 5–9.

The main source of cfDNA in healthy subjects is apoptotic cells but, the cancer cells release longer DNA fragments resulting from necrosis, autophagy, or mitotic catastrophe 8.

The ratio of cancer cell-derived DNA and normal cells-derived DNA called DNA integrity index was found to be increased in cancer patients and may be considered as a malignancy indicator 8,9.

The association between plasma cfDNA concentration and TNBC has not been fully elucidated. The aim of the present study was to investigate the plasma cfDNA concentration and integrity index in breast cancer patients focusing on triple negatives compared with non-triple negative counterparts and normal control.

## Materials and Methods

### Sample collection

The peripheral blood of 96 patients [46 Triple Negative (TN) and 50 non-TN breast cancer patients] from Imam-Khomeini Hospital (Tehran, Iran) and 50 unaffected female blood donors were taken. The patient inclusion criteria were the histopathological diagnosis of ductal carcinoma and availability of Immunohisto-Chemistry (IHC) results for HER-2, ER, PR status and other pathologic diagnostic information (Table 1). Receiving chemotherapy or radiotherapy before recruitment and any history of familial breast disease or malignancy were considered as exclusion criteria.

**Table 1.** Demographic and histoclinical characteristics of breast cancer patients and normal controls

	**Patient N (%)TNBC**	**Patient N (%)non-TNBC**	**Control N (%)**
**Number**	46	50	50
**Age (years)**			
Mean	48.6±11.5	41.5±10.4	48.5±16.4
Range	26–79	25–82	25–80
**Stage at diagnosis**			
Stage II	30(65.2)	30(60%)	
Stage III	12(26.1%)	16(32%)	
Stage IV	4 (8.7%)	4 (8%)	
**Lymph node status**			
N0	24(52.2%)	20 (40%)	
N+	22(47.8%)	30(60%)	
**Distance metastasis**			
Yes	4 [bone] (8.7%)	4[2bone, 2 lung] (8%)	
No	42 (91.3%)	46(98%)	

ER=estrogen receptor, PR=progesterone receptor, TNBC: triple negative breast cancer (ER-, PR-, HER2-), N= number.

The study was approved by the Ethical Committee of the National Institute of Genetic Engineering and Biotechnology (NIGEB) based on the Helsinki declaration. All individuals signed an informed consent to participate in the study.

### Plasma cfDNA extraction

Peripheral blood (10 *ml* in ethylene-diamine-tetraacetic acid) was obtained and the plasma was separated by two sequential centrifugations at 1000 *g*, 4*°C*. The plasma DNA was extracted using the QIAmp DNA Blood Midi Kit (Qiagen, Hiden, Germany) according to the manufacturer’s instructions.

### Plasma DNA quantification and integrity test

Quantification and qualification of plasma DNA were assessed by quantitative real-time PCR (QRTPCR) by ABI 7500/7500 fast real-time system (CA, USA) using human *β-actin* gene as a reference gene. The standard curve was constructed with the DNA dynamic range of 0.01–100 *ng*. Each PCR reaction mixture consisted of 10 *μl* master mix Applied Biosystems™ SYBR™ Green PCR Master Mix (ABI, CA, USA), 1.0 *μl* each primer (0.4 *mM*), 2 *μl* water, and 6 *μl* of extracted DNA. The thermal cycling conditions comprised cycles at 95*°C* for 10 *min* and 40 cycles at 95*°C* for 10 *s* and at 60*°C* for 60 *s* followed by cycles at 72*°C* for 60 *s* in duplicate. Sample DNA concentration was extrapolated from the standard. The OVCAR3 cell line was used as a control, and a negative no template control was included in each run. DNA integrity index was calculated as the ratio between the 394 and 99 *bp* amplicons of the *β-actin* gene. The primer sequences were as follows:

Common forward primer: 5′-CCACACTGTGCCCATCTACG-3′, Reverse primer (β-actin 99 *bp*): 5′AGGATCTTCATGAGGAGTCAGTCAG-3′, Reverse primer (β-actin 394 *bp*): 5′-TTAGCTTCCACAGCACAGCC-3′.

### Statistical analysis

Data were assessed with SPSS (Version 16) software using Mann Whitney and Kruskal Wallis tests. Results were expressed as means±standard deviation, and the p-value less than 0.05 was considered statistically significant. The predictive capability (diagnostic performance) of each biomarker was investigated by means of the area under the ROC (Receiver-Operating Characteristics) curve (AUC).

## Results

### cfDNA concentration and integrity index in breast cancer patients

**Figure 1.**
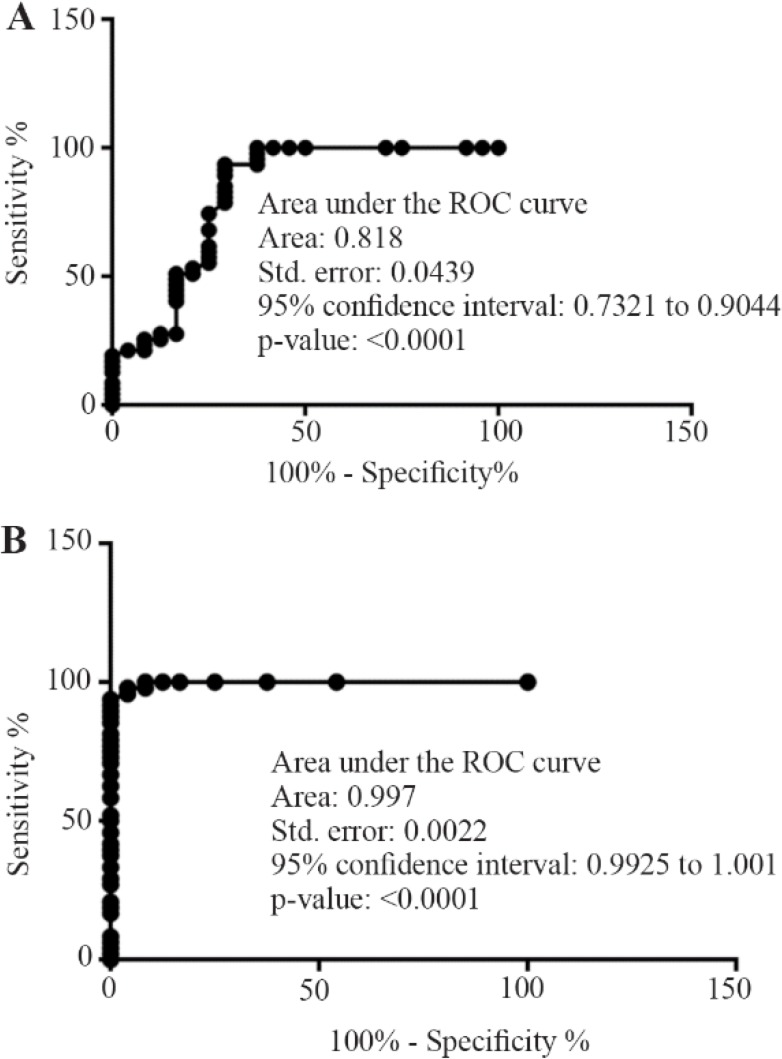
Receiver-operating characteristics plots from the comparison of normal subjects *vs*. breast cancer group focusing on cfDNA concentration(A) and integrity index 394/99 (B).

The cfDNA concentration and integrity index were both significantly elevated in breast cancer patients compared with control (p<0.001). The ROC curve analysis confirmed its possible diagnostic effect (Figure 1).

### Plasma cfDNA concentration and integrity index in TNBC, non-TNBC, and control

**Figure 2.**
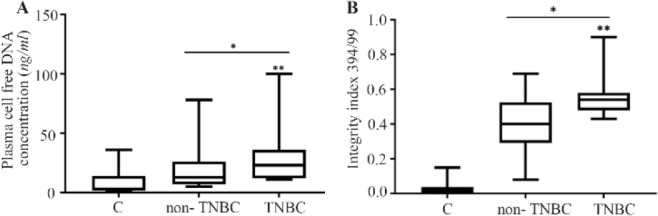
The cfDNA concentration (A) and integrity index (B) in triple negative breast cancer (TNBC) patients compared with non-triple negatives and normal control. * compared with normal control, Mann-Whitney test, p<0.001. ** TNBC compared with non-TNBC and normal control, Kruskal-Wallis test, p*<*0.01. TN: Triple negative breast cancer.

The data revealed the significant increase of plasma cfDNA concentration and integrity index in cancer patients both in TNBC and non-TNBC compared with normal control (p<0.001) (Figure 2, Table 2). The quality and quantity of cfDNA were significantly higher in TNBC group compared with non-TNBC (p< 0.01).

**Table 2.** Cell free DNA concentration in plasma samples of breast cancer and normal control groups

**Sample type (N)**		**cfDNA (*ng/ml*)**	***β-actin* 394 (*ng/ml*)**	**Integrity index 394/99**
**Normal control (50)**				
	Median	3.07	0.11	0.02
	Range	0.6–32	0–1.2	0–0.15
**Non-TNBC (50)**				
	Median	13.22	7	0.4
	Range	5.12–78	0.87–38.5	0.08–0.69
**TNBC (46)**				
	Median	23	11.2	0.55
	Range	11–100	6–56	0.43–0.9

TNBC: triple negative breast cancer (ER-, PR-, HER2 -).

### Quality and quantity of plasma cfDNA concentration focusing on nodal involvement and cancer stages

**Figure 3.**
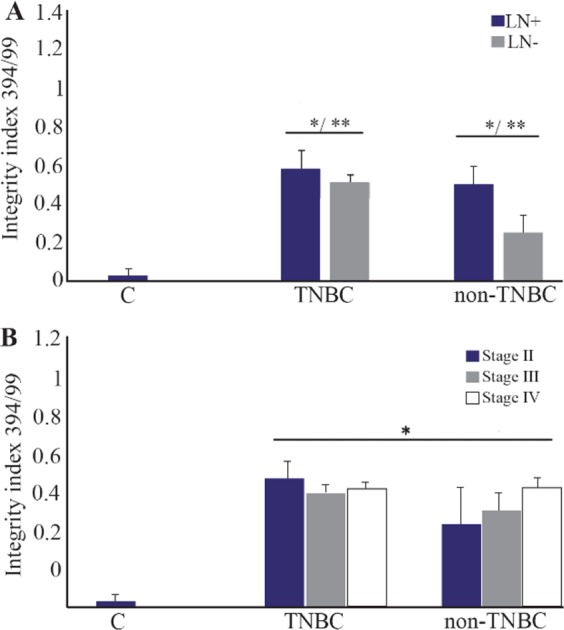
The cfDNA integrity index in TNBC and non- TNBC groups based on lymph node involvement (A) and breast cancer stages (B). * compared with normal control, Mann-Whitney test, p<0.001. ** LN+ compared with LN-, Mann-Whitney test, p*<*0.01. TN: Triple negative breast cancer. LN: Lymph node

The Lymph Node (LN) positive groups, as well as stage IV, showed significantly higher plasma cfDNA concentration (Table 3) and integrity index (Figure 3) in both TNBC and non -TNBC.

**Table 3.** Comparison of cfDNA concentration in triple negative breast cancer, non-triple negative breast cancer and control group based on nodal involvement and cancer stages

**Sample**	**Mean±SD**	**Range**	**Median**	**p-value**
**Control**	8.93±10.25	(0.6–32)	3.07	
**TN/LN+**	45.3±28	(21.5–100)	23	^*^/^**^
**TN/LN**	18.3±7.4	(11–36)	17	^*^
**Non-TN/LN+**	29.6±20.8	(11–78)	19	^*^/^**^
**Non-TN/LN**	7±1.4	(5.12–9.8)	6.85	
**TN/stage II**	19.1±6.8	(11–36)	22	^*^
**TN/stage III**	44±24.2	(23–87)	34	^*^
**TN/stage IV**	84±16	(68–100)	84	^*^/^**^
**Non-TN/stage II**	9±3.4	(5.1–16.76)	8	^*^
**Non-TN/stage III**	29.5±14.5	(13.54–58)	27.28	^*^
**Non-TN/stage IV**	70.5±7.5	(63–78)	70.5	^*^/^**^

* Compared with normal control, Mann-Whitney test, *p*<0.001.

** Compared with the other counterpart groups and control, Kruskal-Wallis test, p<0.05.

TN: Triple negative breast cancer, LN: Lymph node.

## Discussion

The cfDNA levels in plasma/serum seem to be an important universal malignancy marker besides therapy response biomarker in several tumor entities 10–13. The association of cfDNA concentration with necrosis and apoptosis of cancer cells in the tumor microenvironment was reported. Numerous cancer-specific alterations, such as methylation, allelic imbalances, and mutations have been found in blood cfDNA 13. These findings have attracted much attention to the potential use of elevated concentration of circulating DNA as a tumor marker.

It was previously reported that apoptotic cfDNA is fragmented into 180–200 *bp*, whereas cfDNA from the necrotic origin is of higher molecular weight 14. Our data revealed that the higher cfDNA concentration and integrity index were observed in breast cancer patients compared with normal control group. The ROC curve analysis showed the significant diagnostic power of cfDNA concentration and integrity index in breast cancer patients.

The blood cfDNA concentration has been reported as the potential screening and diagnostic biomarker in various cancers such as breast 5,12,15, lung 8,16, renal 17 gastric 18, colorectal 7, and head and neck cancer 11 but population-based standardization of test methods is required prior to clinical use. In the present study, for the first time, the plasma cfDNA concentration and integrity index in TNBC patients was compared with non-TNBC counterparts in a group of Iranian breast cancer patients. The data showed that the mean of plasma cfDNA concentration quantified by measuring *β-actin* gene amplification was significantly higher in both breast cancer groups including triple negative and non-triple negatives compared with normal control. The mean of plasma cfDNA concentration was significantly higher in triple negative breast cancer group compared with non-triple negative counterparts. This higher concentration was associated with higher cancer stages and lymph node involvement. The highest level of plasma cfDNA concentration was observed at stage IV and LN-positive patients. The quantification of two amplicons of the *β-actin* gene with different lengths (99 and 394 *bp*) was used to evaluate the integrity index 394/99. The integrity index was elevated in TNBC compared with non-TNBC as well as lymph node positive patients but there were no statistically significant differences in cfDNA integrity index among different cancer stages. Being triple negative, involving the lymph nodes and having higher stages of the disease, all are the signs of poor prognosis and invasive characteristics of tumors. It could be concluded that higher plasma cfDNA concentration and integrity were associated with more invasive characteristics in cancer. Our data was somehow in line with a study in Egyptian breast cancer patients that reported the higher percentage of cfDNA as well as long cfDNA fragments in breast cancer patients than controls and correlated with higher cfDNA concentration and integrity with HER2 positivity, metastasis and non-treatment response 19. Lo *et al* provided another explanation of cfDNA fragmentation 20. They stated that the plasma DNA molecules showed a predictable fragmentation pattern due to nuclease activity which had been related to the progression of several cancers 20.

The high cfDNA concentration in cancer patients compared with normal individuals may be due to the reason that in cancerous tissues in spite of normal physiologic condition, most of the released DNA from apoptotic and necrotic cells are not removed by macrophages efficiently 21. The main source of circulating cf-DNA in healthy individuals is through apoptosis (mostly short fragments), whereas in cancer patients, it results from both apoptosis and necrosis (mostly long fragments) 22.

## Conclusion

In conclusion, the elevated cfDNA concentration and integrity index in breast cancer patients was compared with normal control and significant difference observed between TNBC and non-TNBC may be considered as a possible effective non-invasive prognostic and diagnostic molecular marker in breast cancer.
